# Cardiovascular Complications in Lupus Patients in the Aseer Region, Saudi Arabia

**DOI:** 10.7759/cureus.43501

**Published:** 2023-08-15

**Authors:** Nouf A Alhammadi, Hanan Alqahtani, Leinah H Alshahrani, Saif A Al Qahtani, Amar A Al Qahtani, Amnah Alharthi, Lama A Asiri, Mashael A Abu Aqil

**Affiliations:** 1 Rheumatology, King Khalid University, Abha, SAU; 2 Rheumatology, Aseer Central Hospital, Abha, SAU; 3 Medicine, King Khalid University, Abha, SAU; 4 General Practice, King Fahad Medical City, Riyadh, SAU

**Keywords:** constitutional symptoms and sle, saudi arabia, sle, systemic lupus erythematosus, cardiovascular complications

## Abstract

Background: Systemic lupus erythematosus (SLE) is an autoimmune disease with high morbidity. The objective of this study was to investigate the prevalence and types of cardiovascular complications among patients with SLE in the Aseer region of Saudi Arabia.

Method: This study is retrospective record-based research conducted at Aseer Central Hospital in the Aseer region from 2020 to 2022. We conducted a comprehensive review of the medical records of patients.

Results: Out of the 189 patients diagnosed with SLE, 18.0% (34 out of 189) experienced cardiovascular complications. Of the patients who experienced cardiovascular complications, around two-fifths (15/34) fell between 35 and 44 years (44.12%), females represented (31/34) 91.20%, the majority were nonsmokers (32/34) 94.0%, (6/32) 17.65% were diabetic, (17/34) 50.0% had hypertension, and (13/34) 38.24% had vasculitis. Pericarditis and myocarditis are seen in (5/34) 14.7% of each of the cases, endocarditis accounts for (4/34) 11.7% of the cases, a myocardial infarction occurs in (7/34) 20.6% of patients, coronary artery disease is prevalent in (14/34) 38.2% of cases, and valvular lesions at (5/34) 14.7%.

Conclusions: Understanding the prevalence of cardiovascular complications in SLE patients is crucial for healthcare providers to tailor treatment plans and preventive measures to address this aspect of the disease.

## Introduction

Systemic lupus erythematosus (SLE) is an autoimmune disease with high morbidity. Classically, disease mortality was attributed to renal involvement [1]. SLE predominantly affects young age females, with the disease peaking around middle adulthood in this group. In contrast, SLE tends to appear later in life in men [2]. SLE is a disease known for its ability to impact multiple systems in the body, leading to a diverse range of clinical features. Patients with SLE commonly present with constitutional symptoms like fatigue and fever, as well as mucocutaneous lesions and musculoskeletal manifestations, such as arthralgia and arthritis. Additionally, approximately half of SLE patients experience blood and neuropsychiatric disorders, along with the involvement of various other organ systems, including the heart, lungs, eyes, kidneys, and gastrointestinal tract [3].

As immunosuppressive therapy improved, renal-related mortality was reduced [1,4]. Cardiovascular mortality cause of mortality around 30% in the first 5 years after diagnosis [5]. Both stroke and myocardial infarction (MI) were noted to occur more often in premenopausal lupus women compared with their counterparts [1,6]. The average age at which such events occurred in comparison to the general population was younger (49 vs. 69 years) [7]. The risk of cardiovascular disease (CVD) in lupus is not completely accounted for by traditional cardiovascular risk factors [8]. The increased risk is not restricted to MIs and stroke but also includes peripheral arterial disease [9].

Compared to adults without SLE, patients with SLE exhibited statistically significantly higher relative risks (RRs) for various cardiovascular outcomes. The RRs (95% confidence intervals) were as follows: stroke (2.51 [2.03-3.10]; 12 studies), MI (2.92 [2.45-3.48]; 11 studies), CVD (2.24 [1.94-2.59]; eight studies), and hypertension (2.70 [1.48-4.92]; seven studies). While the RRs of diabetes (1.24 [0.78-1.96]; three studies) and metabolic syndrome (MetS) (1.49 [0.95-2.33]; seven studies) were elevated, they did not reach statistical significance. Several attempts have been made to investigate the complications associated with SLE [10].

Response to immunosuppressive treatments differed among studies and medications. In a study on peripheral ischemia in lupus, Erdozain et al. noted that patients with lupus with a normal ankle-brachial index had a higher chance of being on cyclophosphamide. This raised the question that perhaps cyclophosphamide may have been protective of peripheral arterial disease in this studied population. However, hydroxychloroquine did not show this potentially beneficial effect [9]. Yet, other studies have shown a reduction in cardiovascular disease in hydroxychloroquine users [11]. In regard to steroids, a study on all cardiovascular endpoints found that the mean duration of steroid use was correlated with a higher risk [12]. Cardiovascular disease pathogenesis in lupus was explained by either atherosclerosis and/or subclinical vasculitis [13].

There is a scarcity of adequate studies on SLE epidemiology in the Kingdom of Saudi Arabia. The prevalence of SLE in Saudi Arabia has been estimated to be 19.28 cases per 100,000 population [14]. Among pediatric patients with SLE, the prevalence of cardiac manifestations was found to be 47.8%. Valvular heart diseases were the most common (34.8%), followed by pericarditis (13%). Interestingly, cardiac involvement was silent in approximately 36.4% of cases, showing no apparent symptoms, while in 9.1% of cases, it presented as the initial symptom of SLE [15]. The objective of this study was to investigate the prevalence of cardiovascular complications among patients with SLE in the Aseer region of Saudi Arabia. The study aims to identify the types of cardiovascular complications that occur in SLE patients in this specific geographic area.

## Materials and methods

This study is retrospective record-based research conducted at Aseer Central Hospital in the Aseer region from 2020 to 2022. In this study, a total of 189 patients with lupus were screened for diabetes mellitus, hypertension, vasculitis, atherosclerosis, coronary artery disease, and peripheral arterial disease.

The inclusion criteria for participants are being at least 18 years old and residing in the Asser region, being diagnosed with SLE, and having cardiovascular complications. Patients younger than 18 and living outside the Asser region were excluded from the study. A datasheet is designed to collect personal characteristics, chronic diseases, cardiac disease diagnoses, valvular disease, vasculitis, carotid artery disease, received medications, and co-morbidities associated with SLE patients.

In this study, we conducted a comprehensive review of the medical records of SLE patients. We focused on traditional cardiovascular risk factors such as tobacco use, diabetes mellitus, hypertension, dyslipidemia, and obesity. In addition to these data, we also collected information about any immunomodulatory disease-modifying agents administered to the study cohort. These agents included steroids, azathioprine, mycophenolate mofetil, rituximab, cyclophosphamide, belimumab, and hydroxychloroquine. The researchers used keywords such as “SLE,” “cardiovascular,” and “complications” to identify the relevant patients in the medical record system. Cardiac complications were diagnosed through the use of the following key diagnostic methods: the electrocardiogram (ECG), echocardiogram (ECHO), and blood tests - specifically, measuring cardiac enzymes such as troponin - and ECHO.

Statistical analysis

In this study, we utilized the Statistical Package for the Social Sciences (SPSS; IBM Corp., Armonk, NY) version 26 for statistical analysis. Categorical variables were expressed in counts and percentages. Microsoft Excel was used to create the figures for this study. The cross table was used to describe the association between dyslipidemia and age group. The Monte Carlo test was used to assess this association. A P-value below 0.05 was considered significant.

Ethical approval

Ethical approval was obtained from the General Directorate of Health Affairs in Madinah. As per the IRB's guidelines based on local regulations, written consent was obtained from all respondents. The study adhered to the principles of the Declaration of Helsinki.

## Results

Regarding age, around two-fifths fell between 35 and 44 years (44.12%) and the least in the below 18 years and 55 years and above categories, each accounting for 5.88% of the sample. Gender distribution shows a higher proportion of females (91.20%) compared to males (8.80%). The majority of patients did not smoke (94.10%) or had diabetes (82.35%). Hypertension was evenly split, with 50.00% of patients having it and the other half not. A notable percentage of patients (35.29%) had a family history of coronary heart disease. Regarding other health conditions, the presence of dyslipidemia was observed in 23.53% of patients, while anemia and vasculitis were found in 38.24% of cases each. Coronary artery disease was less prevalent (2.94%), while nearly three-fourths of patients (70.59%) do not have atherosclerosis. Steroids were the most used medication at 58.82%, followed by Azathioprine (23.53%), Cyclophosphamide (2.94%), and Hydroxychloroquine (67.65%). The mean duration of having SLE was 3.7 ± 0.9 years while the mean duration of receiving treatment for SLE was 2.1 ± 0.9 years (Table 1). There was a significant difference in the prevalence of dyslipidemia across different age groups (p = 0.017) (Table 2).

**Table 1:** Demographic and health profile of systemic lupus erythematosus patients with cardiovascular complications

Studied variables		Number	Percentage
Age	Below 18 years	2	5.88
	From 25 to 34 years	7	20.59
	From 35 to 44 years	15	44.12
	From 45 to 54 years	8	23.53
	55 years and above	2	5.88
Sex	Female	31	91.20
	Male	3	8.80
Smoker	yes	2	5.90
	no	32	94.10
Diabetes	No	28	82.35
	Yes	6	17.65
Hypertension	No	17	50.00
	Yes	17	50.00
Family history of coronary heart disease	Yes	12	35.29
	No	22	64.71
Dyslipidemia	No	24	70.59
	Yes	8	23.53
Anemia	No	21	61.76
	Yes	13	38.24
Vasculitis	No	21	61.76
	Yes	13	38.24
Coronary artery diseases	No	33	97.06
	Yes	1	2.94
Atherosclerosis	No	24	70.59
	Yes	10	29.41
Medications	Steroid	20	58.82
	Azathioprine	8	23.53
	cyclophosphamide	1	2.94
	Hydroxychloroquine	23	67.65
Duration of systemic lupus erythematosus	3.7 ± 0.9		

**Table 2:** Prevalence of dyslipidemia among patients with SLE across different ages SLE: Systemic lupus erythematosus

		Below 18 years		From 25 to 34 years		From 35 to 44 years		From 45 to 54 years		55 years and above	
Dyslipidemia	No	2	7.40%	8	29.60%	11	40.70%	4	14.80%	2	7.40%
	Yes	0	0.00%	0	0.00%	4	50.00%	4	50.00%	0	0.00%
	P = 0.017										

**Figure 1:**
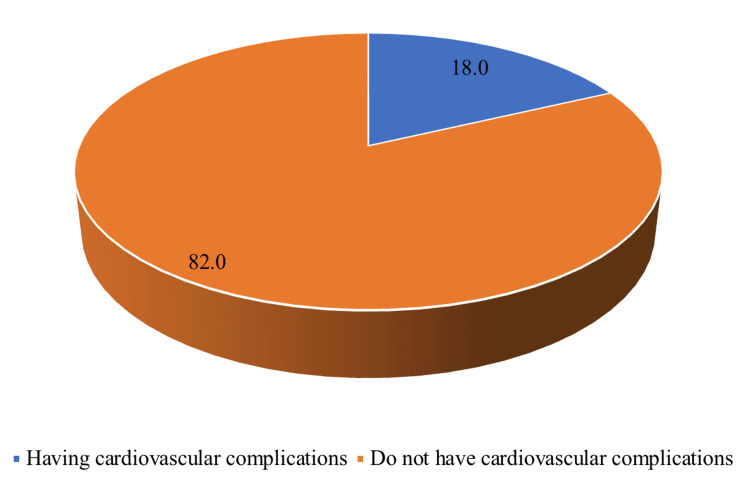
Prevalence of cardiovascular complications among patients with systemic lupus erythematosus

Out of the 189 patients diagnosed with SLE, 18.0% (34 out of 189) experienced cardiovascular complications, as shown in Figure 1.

Table 3 presents the prevalence of different cardiovascular complications in patients with SLE. Pericarditis and myocarditis were seen in 14.71% each of the cases, endocarditis accounted for 11.76% of the cases, MI occurred in 20.59% of patients, coronary artery disease was prevalent in 38.24% of cases, and valvular lesions, at 14.71%.

**Table 3:** Types of cardiovascular complications among patients with SLE

Complication	Number	Percentage
Pericarditis	5	14.71
Myocarditis	5	14.71
Endocarditis	4	11.76
Myocardial infarction	7	20.59
Coronary artery diseases	13	38.24
Valvular lesion	5	14.71

## Discussion

SLE is a complex autoimmune disease that can manifest differently in various patients, affecting multiple organs [16,17]. In this study, our objective was to investigate the prevalence of cardiovascular complications among patients with SLE by collecting data from a single center. Our findings revealed that the overall incidence of cardiovascular complications in the SLE patient population was 18.0%. On the other hand, based on the findings of Danielle et al. [13], the prevalence of cardiovascular complications among 6.5%. However, this indicates that cardiovascular involvement is relatively common in individuals with SLE.

In our study, we acknowledge that the relatively small sample size limited our ability to assess whether gender was related to the incidence of cardiovascular complications among patients with SLE. Nevertheless, it is widely recognized that SLE exhibits a higher prevalence in women compared to men, although male patients often experience a more rapid and severe disease course. This intriguing observation has sparked extensive research aimed at understanding the sex-based differences in the development and pathogenesis of SLE in patients. Specifically, it was noted that male patients with SLE tend to have a higher occurrence of renal involvement compared to women [16,17]. So that investigations with larger sample sizes are warranted to elucidate the potential relationship between sex and cardiovascular complications in SLE and to better understand the complex nature of the disease and its varied manifestations based on gender. In our study, we observed that the majority of cardiovascular complications in patients with SLE occurred among those aged 35 to 45 years. This age group showed a higher prevalence of such complications compared to other age categories.

In our study, a substantial proportion of patients who developed cardiovascular complications in the context of SLE had pre-existing chronic conditions. Specifically, we found that 17.5% of patients had diabetes, 38.24% had vasculitis, 23.53% had dyslipidemia, and 50.0% had hypertension. These risk factors have been widely addressed in previous studies [18-20]. These findings suggest that the presence of these co-existing chronic diseases may contribute to an increased risk of developing cardiovascular complications in SLE patients. Managing and monitoring these comorbidities alongside SLE is crucial to improve overall health outcomes and reduce the risk of cardiovascular events.

In our study, we observed several cardiovascular complications among patients with SLE. Pericarditis and myocarditis were each reported in 14.71% of the cases, endocarditis accounted for 11.76% of the cases, and MI occurred in 20.59% of patients. Additionally, coronary artery disease was prevalent in 38.24% of cases, and valvular lesions were observed in 14.71% of the patients. The most commonly affected valves were the mitral followed by the aortic valve. Danielle et al. [13] found that 35% of those who had cardiovascular complications had experienced MI, 10 (50%) had experienced stroke and four (20%) had peripheral ischemia. These findings underscore the diverse nature of cardiovascular complications that can occur in the context of SLE, affecting various components of the cardiovascular system. It is essential for clinicians to be vigilant in monitoring and managing these complications to ensure timely intervention and improve patient outcomes.

Limitations and strengths

This study has some notable limitations that should be acknowledged. Firstly, the small sample size of included patients may limit our ability to perform robust inferential statistics to study the associations between cardiovascular events and other covariates. This could potentially affect the generalizability of our findings to a larger SLE population. Secondly, the retrospective nature of data collection from medical records may introduce recording and recall biases, as the accuracy and completeness of recorded information can vary. Additionally, we were unable to collect real-time data, which might have affected the accuracy of certain clinical details. Finally, we did not perform coronary computed tomography angiography (CTCA) in our evaluation. Instead, our diagnostic approach relied on ECG, ECHO, and measurements of cardiac enzymes. Despite these limitations, it is essential to recognize that this study represents the first attempt to assess the prevalence of cardiovascular complications among patients with SLE in Saudi Arabia. This pioneering effort sheds light on the cardiovascular burden experienced by SLE patients in our region, providing valuable insights for further research and clinical management. Future studies with larger sample sizes and prospective designs are warranted to corroborate and expand upon our findings, allowing for more comprehensive investigations into the associations between SLE and cardiovascular outcomes.

## Conclusions

This research highlights the importance of screening for cardiovascular risks in individuals with SLE as those who develop complications may require specific attention and care to mitigate the impact on their health. Understanding the prevalence of cardiovascular complications in SLE patients is crucial for healthcare providers to tailor treatment plans and preventive measures to address this aspect of the disease. Further research and investigations are warranted to delve deeper into the specific risk factors contributing to cardiovascular complications in SLE and to identify effective strategies for early detection and management. Overall, the findings underscore the need for comprehensive care and a multidisciplinary approach when dealing with SLE patients, with close consideration of cardiovascular health in the management of the disease.
